# The Mental Health of Veterinarians in Türkiye: A Prevalence Study

**DOI:** 10.1155/da/2386148

**Published:** 2026-08-28

**Authors:** Ahmet Metin, Çağrı Çağlar Sinmez, Ali İlteriş Aykun, Eyüp Sabır Erbiçer

**Affiliations:** ^1^ Department of Guidance and Counseling, Erciyes University, Kayseri, Türkiye, erciyes.edu.tr; ^2^ Department of History of Veterinary Medicine and Deontology, Erciyes University, Kayseri, Türkiye, erciyes.edu.tr; ^3^ Department of Guidance and Counseling, Siirt University, Siirt, Türkiye, siirt.edu.tr

**Keywords:** anxiety, depression, prevalence, resilience, stress, Turkey, veterinarians

## Abstract

**Background:**

Veterinarians have been identified as a high‐risk occupational group for mental health problems; however, evidence regarding their mental health status in Türkiye remains limited. This study aimed to examine the prevalence of depression, anxiety, stress, and resilience among veterinarians in Türkiye.

**Methods:**

A cross‐sectional study was conducted with 821 veterinarians (M_age_ = 34.94, SD = 8.60; range 19–65) working in both public and private sectors in Türkiye. Participants were recruited using a stratified sampling method based on sex, employment sector, and geographical region. Data were collected using the Depression, Anxiety, and Stress Scale (DASS‐21) and the Brief Resilience Scale (BRS). Independent samples *t*‐tests, Pearson correlation analyses, and chi‐square tests were used to analyze the data.

**Results:**

The findings indicated that 34% of participants reported depression, 35% anxiety, and 39% severe or extremely severe stress levels. Approximately 35% of veterinarians were found to have low resilience. Female veterinarians reported significantly higher levels of depression, anxiety, and stress, as well as lower resilience, compared to males. In addition, veterinarians working in the public sector exhibited significantly lower levels of depression, anxiety, and stress than those working in the private sector.

**Conclusion:**

The results indicate that mental health problems are prevalent among veterinarians in Türkiye and vary across demographic and occupational groups. These findings contribute to the limited evidence base on veterinary mental health in middle‐income country contexts.

## 1. Introduction

Mental health problems, including depression, anxiety, and stress, constitute a major public health concern with far‐reaching consequences for individuals’ daily functioning, occupational performance, and overall well‐being [1]. These challenges are not uniformly distributed across professions; rather, certain occupational groups are disproportionately exposed to psychological risks due to the nature of their work. Among these, veterinarians have consistently been identified as a high‐risk group for adverse mental health outcomes [2]. The veterinary profession involves sustained exposure to multiple stressors, including responsibility for animal health and welfare, public health obligations, ethical dilemmas related to life‐and‐death decisions, financial constraints affecting treatment, heavy workloads, and high client expectations [3, 4].

A growing body of international research demonstrates that veterinarians experience significantly elevated levels of psychological distress compared to those in the general population. Evidence from Europe, North America, and Australia indicates higher prevalence rates of depression, anxiety, stress, and suicidal ideation among veterinary professionals [2, 5, 6]. For example, studies conducted in the United Kingdom and Finland have documented substantial levels of psychological distress and burnout, particularly among female veterinarians and those working in demanding clinical settings [2, 7]. Similarly, research from Germany has highlighted increased suicide risk and elevated stress levels among practicing veterinarians [8, 9]. Comparable findings have been reported in North America and Australia, where veterinarians exhibit higher rates of depression, stress, and suicide compared to population norms [5, 10, 11].

More recent studies further reinforce and extend this evidence by highlighting the severity and complexity of mental health problems within the veterinary profession across diverse contexts. Veterinarians have been shown to exhibit significantly higher levels of depression, anxiety, and insomnia symptoms compared to the general population, with risk factors including being female, long working hours, and limited professional experience [12]. Occupational stressors such as financial concerns have been identified as particularly critical determinants of mental health, even when not perceived as the most burdensome aspects of the profession [13]. Evidence from Hong Kong indicates substantial prevalence rates of depression, anxiety, and suicide risk, with burnout emerging as a key predictor of depressive symptoms [14]. Similarly, findings from Portugal demonstrate high levels of severe depression, anxiety, stress, burnout, and suicide risk among veterinary professionals, particularly among those with longer working hours and prior mental health problems [15].

Recent research has also emphasized the role of occupational and structural factors in shaping mental health outcomes. Burnout remains highly prevalent among veterinarians, with work–life imbalance, ethical dilemmas, and long working hours identified as major contributing factors [16]. In addition, elevated psychological stress and overcommitment have been observed among veterinarians, with inadequate compensation and prolonged exposure to stress contributing to an increased risk of burnout and depression [17]. Notably, low reward, high overcommitment, and burnout have been identified as the strongest predictors of depression, suicidal ideation, and suicide risk in veterinary populations [18]. Taken together, these findings indicate that veterinarians across countries consistently experience elevated psychological distress; however, the relative contributions of occupational, economic, and contextual factors vary across settings, underscoring the importance of country‐specific investigations.

To better understand these dynamics, the Job Demands–Resources (JD–R) model provides a useful theoretical framework [19, 20]. According to this model, employe well‐being is shaped by the balance between job demands and job resources. Job demands, such as a high workload, emotional labor, and ethically challenging decision‐making, are associated with increased psychological strain, whereas job resources, including organizational support and access to coping mechanisms, can mitigate these effects. In veterinary practice, the imbalance between high demands and limited resources may contribute substantially to adverse mental health outcomes.

Another important factor associated with mental health is resilience, which has been shown to play a protective role against psychological distress [21–23]. Understanding resilience levels among veterinarians is, therefore, essential for identifying both risk and protective factors influencing mental health outcomes.

## 2. Current Study

Despite the growing body of international research, the mental health of veterinarians remains an issue that requires further systematic investigation across different contexts. Assessing levels of depression, anxiety, and stress is essential for understanding the psychological well‐being of this professional group and may provide a basis for the development of preventive and intervention strategies aimed at protecting their mental health [21]. In this regard, veterinary organizations and the broader professional community are increasingly encouraged to implement structured interventions to improve mental health outcomes and support professional functioning [24]. Therefore, examining the mental health status of veterinarians is not only relevant for individual well‐being but also for sustaining the quality and effectiveness of veterinary services.

In addition to psychological distress, resilience represents a critical determinant of mental health. As a protective factor, resilience has been shown to buffer the negative effects of occupational stressors and contribute to better mental health outcomes [21–23]. Accordingly, assessing resilience alongside depression, anxiety, and stress provides a more comprehensive understanding of both risk and protective mechanisms underlying veterinarians’ mental health.

Although numerous studies have examined the mental health of veterinarians in various countries (e.g., [5, 8, 10, 25, 26]), there is, to our knowledge, no study that comprehensively evaluates depression, anxiety, stress, and resilience among veterinarians in Türkiye. This lack of data limits the ability to compare the mental health status of Turkish veterinarians with that of their counterparts in other countries. Addressing this gap is particularly important given the distinctive occupational characteristics of veterinary practice, which involve sustained exposure to high workload, emotional labor, ethical dilemmas, and client‐related pressures—factors that have consistently been associated with psychological distress in the global literature [5, 10, 25].

Furthermore, empirical evidence from middle‐income countries remains limited despite the likelihood that socioeconomic and structural factors may significantly shape mental health outcomes. Focusing on veterinary physicians in Türkiye, therefore, provides an opportunity to extend the existing literature by incorporating a context characterized by ongoing economic pressures, demanding working conditions, and relatively limited access to occupation‐specific mental health resources. Within such a context, constraints related to organizational support and access to psychological resources may further restrict the availability of job resources.

From a theoretical perspective, the JD–R model offers a useful framework for interpreting these dynamics as prolonged imbalance between high job demands and insufficient resources may increase vulnerability to depression, anxiety, and stress. Examining veterinarians in Türkiye within this framework may therefore contribute to a more nuanced understanding of how contextual and occupational factors interact to influence mental health outcomes.

In order to capture this complexity, data were collected from veterinarians working across both public and private sectors, encompassing a wide range of organizational contexts and working conditions. Participants were recruited from different geographical regions of Türkiye, with the sampling strategy designed to reflect variability in occupational settings and exposure to stressors, thereby enhancing the contextual relevance and interpretability of the findings.

Therefore, the present study aims to address the existing gap in the literature by examining the prevalence of depression, anxiety, stress, and resilience among veterinarians in Türkiye. In addition, the study seeks to explore whether these outcomes differ according to sex, employment status, and geographical location. By providing context‐specific evidence, this study aims to contribute to the global understanding of veterinary mental health and to inform the development of targeted, evidence‐based interventions. This study provides the first comprehensive prevalence data on depression, anxiety, stress, and resilience among veterinarians in Türkiye, contributing to the limited evidence base from middle‐income countries.

The following research questions were addressed in the present study:

1. What is the prevalence of depression, anxiety, stress, and resilience among veterinarians in Türkiye?
2. Do these variables differ according to sex, employment status, and geographical location?

## 3. Methodology

### 3.1. Participants and Procedure

A stratified recruitment approach was used, with an online questionnaire administered to veterinarians in Türkiye between August 10 and November 30, 2022. The study was reported in accordance with the STROBE guidelines for cross‐sectional studies [27]. Data were collected anonymously, and informed consent was obtained electronically prior to participation. Ethical approval was granted by the Social and Human Sciences Ethics Committee of X University. The target population consisted of veterinarians working in the public and private sectors in Türkiye (*N* = 35,898; [28, 29]. A stratified random sampling approach was employed based on sex, employment sector (public vs. private), and geographical region. Participants were selected proportionally within each stratum to reflect the distribution of veterinarians across Türkiye.

Eligibility criteria included (a) being aged 18 years or older, (b) currently working as a veterinarian in Türkiye, (c) ability to read and understand Turkish, and (d) having access to the internet to complete the online questionnaire. The response rate could not be precisely calculated due to the online survey’s distribution method.

Among the participants, 206 (25.1%) were female and 615 (74.9%) were male. The mean age was 34.94 years (SD = 8.60). Of the sample, 196 (23.9%) were employed in the public sector and 625 (76.1%) in the private sector. Participants were distributed across geographical regions, with the highest representation from the Marmara (27.2%) and Central Anatolia (30.5%) regions. Detailed information is shown in Table 1.

**Table 1:** Stratified sampling process.

Stratified variables	Universe (*n*)	Universe (%)	Sample (*n*)	Sample (%)
Gender				
Female	7025	24.3	206	25.1
Male	28,873	75.7	615	74.9
Work status				
Public	7892	21.9	196	23.9
Private	28,006	78.0	625	76.1
Geographical region				
Marmara	9173	25.5	223	27.2
Aegean	6281	17.4	115	14.0
Black Sea	3386	9.43	66	8.0
Central Anatolia	7126	19.8	250	30.5
Southeastern Anatolia	2362	6.5	37	4.5
Eastern Anatolia	3508	9.7	44	5.4
Mediterranean	4062	11.3	86	10.5
Total	35,898	100	821	100

### 3.2. Screening Tools

#### 3.2.1. Personal Information Form

The background information form developed by the research team consists of brief demographic and occupational details, including age, sex, the main type of work in the profession, and data on the region in which to live in Türkiye.

#### 3.2.2. Depression, Anxiety, and Stress Scale (DASS‐21)

Depression, anxiety, and stress were assessed using the DASS‐21 [30, 31], which were adapted into Turkish by Sarıçam [32]. It is a widely used self‐report instrument consisting of 21 items rated on a 4‐point Likert scale (0–3). The scale includes three subscales measuring depression, anxiety, and stress. Subscale scores were multiplied by two to obtain comparable scores with the original 42‐item version. Based on the recommended cut‐off scores [30], depression was categorized as normal (0–9), mild (10–13), moderate (14–20), severe (21–27), and extremely severe (28+); anxiety as normal (0–7), mild (8–9), moderate (10–14), severe (15–19), and extremely severe (20+); and stress as normal (0–14), mild (15–18), moderate (19–25), severe (26–33), and extremely severe (34+). The internal consistency coefficients in the present study were high, with Cronbach’s alpha values of 0.90 for depression, 0.87 for anxiety, and 0.86 for stress.

#### 3.2.3. Brief Resilience Scale (BRS)

Resilience was assessed using the BRS [33], which was adapted into Turkish by Doğan [34] and consists of six items rated on a 5‐point Likert scale. Higher scores indicate greater resilience. Based on recommended cut‐off values, scores were categorized as low, normal, and high resilience. Smith et al. [33] suggested that scores below 3.00 indicate a low level of resilience, scores between 3.00 and 4.30 represent a normal level of resilience, and scores above 4.30 indicate a high level of resilience. The total sum was divided by the total number of questions to obtain the BRS levels among veterinarians. In the present study, the internal consistency coefficient for the BRS was acceptable with a Cronbach’s alpha of 0.82.

### 3.3. Data Collection Process

Data were collected using an anonymous online questionnaire. Participation was voluntary, and informed consent was obtained electronically prior to survey completion. To enhance data integrity, the survey was configured to allow a single response per participant, thereby reducing the likelihood of duplicate entries. The survey link was distributed through verified professional networks and communication groups of veterinarians to limit access to eligible participants.

### 3.4. Statistical Analysis

Data analysis was conducted using SPSS version 28 (SPSS for Windows, Version 28.0; SPSS Inc., Chicago, IL). Prior to the analysis, skewness and kurtosis values were examined to assess the normality of the data distribution. The results showed that the skewness ranged from –0.05 to +0.95, and kurtosis ranged from −0.89 to +0.69. These values indicated that the DASS and BRS scores were normally distributed [35]. Data distribution would not be a concern for the present study. Therefore, Pearson’s correlation was run to examine the relationships between DASS‐21 (i.e., depression, anxiety, and stress) and resilience. An independent sample *t*‐test was also conducted to investigate the differences in depression, anxiety, stress, and resilience scores by sex and sector. Additionally, a chi‐square test of independence was run to examine the associations between the DASS‐21 (i.e., depression, anxiety, and stress), the BRS level, sex, and sector. Missing data were also minimal and handled using complete‐case analysis (i.e., listwise deletion), and all analyses were performed with a final sample of 821 participants.

## 4. Results

A Pearson’s correlation was run to examine the relationship between depression, anxiety, stress, and resilience. The means, SDs, and correlations for the study variables are presented in Table 2. The results indicated that resilience was statistically significant and negatively associated with depression (*r* = −0.57, *p* < 0.01), anxiety (*r* = −0.55, *p* < 0.01), and stress (*r* = −0.55, *p* < 0.01).

**Table 2:** Descriptive statistics and correlations for study variables.

Variables	M	SD	*α*	1	2	3	4	5
1. Depression^a^	17.81	11.79	0.90	—	—	—	—	—
2. Anxiety^a^	14.77	10.57	0.87	0.74 ^∗∗^	—	—	—	—
3. Stress^a^	21.37	10.67	0.86	0.78 ^∗∗^	0.80 ^∗∗^	—	—	—
4. Resilience	3.24	0.90	0.82	−0.57 ^∗∗^	−0.55 ^∗∗^	−0.55 ^∗∗^	—	—
5. Age	34.94	8.60	—	−0.17 ^∗∗^	−0.14 ^∗∗^	−0.13 ^∗∗^	0.15 ^∗∗^	—

*Note: N* = 821. M = mean value; *α* = Cronbach’s alpha.

Abbreviation: SD, standard deviation.

^a^Subscale of the DASS‐21.

^∗^ *p* < 0.05.

^∗∗^ *p* < 0.01.

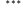
 *p* < 0.001.

The prevalence of depression, anxiety, stress, and resilience levels is presented in Table 3. A substantial proportion of veterinarians reported severe and extremely severe levels of depression (37.4%), anxiety (45.0%), and stress (39.4%). In contrast, 27.6%, 27.8%, and 29.8% of veterinarians were classified in the normal range for depression, anxiety, and stress, respectively. For resilience, 34.7% of veterinarians were classified as having low resilience, whereas 50.5% and 14.7% were classified as having normal and high resilience, respectively.

**Table 3:** Prevalence of depression, anxiety, and stress among veterinarians.

Outcome	Level	*n*	%	95% CI
Depression	Normal	227	27.6	[24.7, 30.8]
	Mild	92	11.2	[9.2, 13.5]
	Moderate	195	23.8	[21.0, 26.9]
	Severe	110	13.4	[11.2, 15.9]
	Extremely severe	197	24.0	[21.2, 27.1]
Anxiety	Normal	228	27.8	[24.8, 30.9]
	Mild	68	8.3	[6.6, 10.4]
	Moderate	156	19.0	[16.5, 21.8]
	Severe	100	12.2	[10.1, 14.6]
	Extremely severe	269	32.8	[29.7, 36.1]
Stress	Normal	245	29.8	[26.8, 33.1]
	Mild	105	12.8	[10.7, 15.2]
	Moderate	147	17.9	[15.5, 20.7]
	Severe	189	23.0	[20.3, 26.0]
	Extremely severe	135	16.4	[14.0, 19.1]
Resilience	Low	285	34.7	[31.5, 38.0]
	Normal	415	50.5	[47.1, 54.0]
	High	121	14.7	[12.5, 17.3]

*Note: N* = 821. Severity levels were classified based on recommended DASS‐21 cut‐off scores.

Differences in depression, anxiety, stress, and resilience scores by sex and work status are presented in Table 4. Female veterinarians reported higher levels of depression, anxiety, and stress than male veterinarians (*p* < 0.001), with small to moderate effect sizes. Female veterinarians also reported lower resilience scores than males (*p* < 0.001, moderate effect size). Veterinarians working in the private sector reported higher levels of depression, anxiety, and stress than those working in the public sector (*p* < 0.05), with small effect sizes. No statistically significant difference was observed in resilience scores between sectors (*p* > 0.05).

**Table 4:** DASS‐21 and BRS scores of veterinarians by demographic variables.

Outcome	Group	*n*	M	SD	*t*	*p*	*d*
Depression	Female	206	20.25	11.39	3.45	<0.001	0.28
	Male	615	16.99	11.83	—	—	—
Anxiety	Female	206	18.57	10.58	6.09	<0.001	0.50
	Male	615	13.50	10.27	—	—	—
Stress	Female	206	24.71	10.04	5.27	<0.001	0.44
	Male	615	20.25	10.65	—	—	—
Resilience	Female	206	2.94	0.88	−5.63	<0.001	0.46
	Male	615	3.34	0.89	—	—	—
Depression	Public	196	16.35	12.34	−2.00	0.046	0.17
	Private	625	18.28	11.59	—	—	—
Anxiety	Public	196	13.22	10.54	−2.36	0.019	0.19
	Private	625	15.26	10.54	—	—	—
Stress	Public	196	19.47	11.16	−2.87	0.004	0.24
	Private	625	21.97	10.45	—	—	—
Resilience	Public	196	3.27	0.97	0.61	0.542	0.05
	Private	625	3.23	0.88	—	—	—

*Note: N* = 821. M = mean value; *d* = Cohen’s *d*. Positive *d* values indicate higher scores in females (or private sector).

Abbreviation: SD, standard deviation.

Associations between categorical levels of depression, anxiety, stress, and resilience and demographic variables are presented in Table 5. Sex was significantly associated with depression level, *χ* ^2^(4) = 11.17, *p* = 0.03; anxiety level, *χ* ^2^(4) = 35.48, *p* < 0.001; stress level, *χ* ^2^(4) = 27.01, *p* < 0.001; and resilience level, *χ* ^2^(2) = 22.59, *p* < 0.001. Females showed higher proportions in the severe and extremely severe categories of depression, anxiety, and stress and a higher proportion in the low resilience category than males. Work status was significantly associated with anxiety level, *χ* ^2^(4) = 9.85, *p* = 0.04. Veterinarians in the private sector showed higher proportions in the severe and extremely severe anxiety categories than those in the public sector. Work status was not significantly associated with depression, stress, or resilience levels (*p* > 0.05).

**Table 5:** Prevalence of depression, anxiety, and stress among veterinarians by gender and sector.

Outcome	Level	Female *n* (%)	Male *n* (%)	*χ* ^2^(df), *p*	Public *n* (%)	Private *n* (%)	*χ* ^2^(df), *p*
Depression	Normal	39 (18.9)	188 (30.6)	*χ* ^2^(4) = 11.17, *p* = 0.03	68 (34.7)	159 (25.4)	*χ* ^2^(4) = 6.99, *p* = 0.14
	Mild	25 (12.1)	67 (10.9)	—	21 (10.7)	71 (11.4)	—
	Moderate	52 (25.2)	143 (23.3)	—	39 (19.9)	156 (25.0)	—
	Severe	34 (16.5)	76 (12.4)	—	26 (13.3)	84 (13.4)	—
	Extremely severe	56 (27.2)	141 (22.9)	—	42 (21.4)	155 (24.8)	—
Anxiety	Normal	30 (14.6)	198 (32.2)	*χ* ^2^(4) = 35.48, *p* < 0.001	66 (33.7)	162 (25.9)	*χ* ^2^(4) = 9.85, *p* = 0.04
	Mild	12 (5.8)	56 (9.1)	—	22 (11.2)	46 (7.4)	—
	Moderate	45 (21.8)	111 (18.0)	—	36 (18.4)	120 (19.2)	—
	Severe	24 (11.7)	76 (12.4)	—	20 (10.2)	80 (12.8)	—
	Extremely severe	95 (46.1)	174 (28.3)	—	52 (26.5)	217 (34.7)	—
Stress	Normal	39 (18.9)	206 (33.5)	*χ* ^2^(4) = 27.01, *p* < 0.001	70 (35.7)	175 (28.0)	*χ* ^2^(4) = 5.84, *p* = 0.21
	Mild	25 (12.1)	80 (13.0)	—	24 (12.2)	81 (13.0)	—
	Moderate	31 (15.0)	116 (18.9)	—	37 (18.9)	110 (17.6)	—
	Severe	63 (30.6)	126 (20.5)	—	39 (19.9)	150 (24.0)	—
	Extremely severe	48 (23.3)	87 (14.1)	—	26 (13.3)	109 (17.4)	—
Resilience	Low	99 (48.1)	186 (30.2)	*χ* ^2^(2) = 22.59, *p* < 0.001	65 (33.2)	220 (35.2)	*χ* ^2^(2) = 0.29, *p* = 0.86
	Normal	87 (42.2)	328 (53.3)	—	102 (52.0)	313 (50.1)	—
	High	20 (9.7)	101 (16.4)	—	29 (14.8)	92 (14.7)	—

*Note: N* = 821. Percentages are column percentages. All expected cell counts were ≥5.

## 5. Discussion

The current study aimed to examine the prevalence of depression, anxiety, stress, and resilience among veterinarians in Türkiye and to explore differences across sex, employment sector, and geographical region. The findings indicate that approximately one in three veterinarians experience severe or extremely severe levels of depression, anxiety, and stress, accompanied by low levels of resilience. In addition, female veterinarians reported higher levels of psychological distress and lower resilience, while those working in the public sector exhibited comparatively lower levels of depression, anxiety, and stress. These results suggest that mental health problems among veterinarians in Türkiye are both prevalent and unequally distributed across demographic and occupational groups, highlighting the importance of contextual and structural factors in shaping psychological outcomes.

### 5.1. Mental Health Issues (Depression, Anxiety, and Stress) and Resilience

Our research findings revealed that approximately one in three veterinarians have severe or extremely severe depression, anxiety, and stress levels, and their resilience levels are low. The findings are consistent with the relevant literature [2–4, 6, 12–15, 36–40]. The mental health of veterinarians is influenced by external factors such as personality traits, sex, client socioeconomic status, client interactions, euthanasia procedure, time since graduation, type of veterinary clinic, unexpected outcomes of clinical cases, medical errors, and patient care responsibility [4, 41–44, 40, 45–52]. However, studies have shown that factors such as being on‐call or working long hours, excessive workload (volume of work), workplace pressure (time per client, number of consultations, time spent in surgery, number of telephone calls), social isolation, inadequate rest/holiday, poor work/home‐life balance [52–54], and administrative duties are the most frequently cited stress factors that negatively affect veterinary mental health [2, 55–57]; Fristschi et al., 2009; [5, 40, 50, 52, 58–61]). Therefore, environmental factors such as workload and job stress may be risk factors for veterinarians [62]. From a theoretical perspective, these findings can be interpreted within the framework of the JD–R model. High job demands, such as workload, emotional labor, and client‐related pressures, combined with limited access to job resources, may increase vulnerability to psychological distress. Recent evidence supports this interpretation by showing that work–life imbalance, ethical dilemmas, and long working hours are major contributors to burnout [16], while overcommitment and inadequate compensation are strongly associated with depression and burnout [17, 18].

Differences across studies may also be attributed to methodological factors, including the use of varying study designs, data collection methods, and measurement tools, which can impact the prevalence rates and symptom severity [63, 64]. In addition, variation in sample characteristics—such as work setting, years of experience, and professional role—has been associated with differences in depression, anxiety, and stress among veterinarians [40, 65]. Together, these factors suggest that mental health outcomes among veterinarians should be interpreted within their specific occupational and cultural context.

In a systematic review, veterinarians’ sources of stress were categorized as biological, psychological, and social. The primary source of stress was client interactions [62]. These workplace factors may lead to physical, psychological, social, behavioral, and organizational problems among veterinarians [66]. Notably, small‐pet and mixed‐practice veterinarians have more anxiety and depression than cattle veterinarians [45, 58], which can be given as an example of the work stress caused by interactions with clients and pets. Another reason may be that novice veterinarians pressure themselves to prove themselves in their careers, which increases stress [37]. Veterinarians with limited professional experience may struggle to cope with professional challenges [37]. Another factor that may cause veterinarians to experience mental health problems may be the COVID‐19 pandemic. The data for the current study were collected in 2022 after the COVID‐19 pandemic. Meta‐analysis studies reveal that the fear of COVID‐19 is negatively correlated with mental health problems such as depression, anxiety, and stress [67, 68]. Therefore, the pandemic may have negatively affected the mental health of veterinarians [38].

### 5.2. Sex and Mental Health

Female veterinarians in the present study reported higher levels of depression, anxiety, and stress, as well as lower levels of resilience, which is consistent with previous findings in both veterinary‐specific and general mental health literature [6, 36, 69]. Recent evidence further indicates that being female, along with long working hours and lower professional experience, is associated with an increased risk of depression and anxiety among veterinarians [12]. In addition, burnout has been shown to be more prevalent among younger and female veterinary professionals, particularly amid work–life imbalance and demanding working conditions [16]. Given that the present study did not directly assess underlying mechanisms, these findings should be interpreted with caution; however, they suggest that sex‐related differences in mental health may reflect differential exposure to occupational demands within veterinary practice rather than factors examined beyond the scope of the current data.

### 5.3. Implications for Practice, Research, and Policy

The findings of the present study have several important implications for research, practice, and policy. First, the high prevalence of depression, anxiety, and stress, together with low levels of resilience, underscores the need for systematic and context‐sensitive mental health interventions for veterinarians in Türkiye. Although the present study did not directly examine the underlying causes of these outcomes, future research should prioritize identifying specific risk and protective factors and exploring veterinarians’ lived experiences to develop more targeted and effective interventions. In this regard, expanding the evidence base through both quantitative and qualitative approaches would provide a more comprehensive understanding of mental health in this population.

From a practice and organizational perspective, improving veterinarians’ mental health is critical not only for individual well‐being but also for the quality and safety of professional practice. Previous research has shown that mental health problems among healthcare professionals may negatively affect clinical performance and increase the likelihood of errors [70, 71], and similar risks have been suggested for veterinarians [72]. Therefore, promoting psychological well‐being should be considered an essential component of professional standards in veterinary practice. Interventions aimed at enhancing resilience are particularly promising as resilience is not a fixed trait but can be developed through targeted training and support [21]. Psychoeducational and mindfulness‐based interventions have been shown to be effective in reducing stress and improving coping capacities [73–76], suggesting that similar programs could be adapted for veterinarians in both the public and private sectors.

At the organizational level, institutional support mechanisms play a key role in promoting mental health. Providing structured training programs, improving communication within institutions, and strengthening professional support systems may enhance both resilience and a sense of belonging among veterinarians [77, 78]. In addition, facilitating access to flexible working arrangements, ensuring adequate rest periods, and promoting work–life balance are likely to support engagement in health‐protective behaviors and access to social support, particularly under high‐workload conditions [57].

At the policy and educational level, there is a need to expand access to mental health services and to integrate preventive mental health strategies into veterinary education and professional development. Evidence from several countries indicates that structured interventions—such as 24/7 support services, mental health training programs, and professional skill development modules—can improve well‐being among veterinary professionals [79]. Implementing similar initiatives within veterinary faculties and healthcare systems in Türkiye may help reduce barriers to mental health support and promote early intervention.

Finally, future research should also examine veterinarians’ help‐seeking behaviors and access to mental health services as stigma and structural barriers may limit the use of available support systems. Generating such evidence would provide a stronger scientific basis for designing and implementing mental health policies tailored to veterinarians’ needs in Türkiye. Overall, addressing mental health through multilevel, evidence‐based strategies has the potential not only to improve individual well‐being but also to enhance the sustainability and effectiveness of veterinary practice.

## 6. Limitations

The findings of this study should be interpreted in light of several methodological and theoretical limitations. Although the study was conducted on a large and diverse sample of veterinarians from both public and private sectors in Türkiye, and the distribution of participants supports a degree of generalizability, certain constraints should be acknowledged. From a sampling perspective, the stratified sampling approach was limited to sex, employment sector (public vs. private), and geographical region. Other potentially relevant variables—such as professional specialization, years of experience, and workplace characteristics—were not included in the stratification process, which may have influenced the representativeness of specific subgroups. In addition, because of the online survey’s distribution method, it was not possible to determine the exact number of individuals who received the survey invitation; therefore, nonresponse bias could not be formally assessed. From a measurement perspective, the assessment of depression, anxiety, stress, and resilience was restricted to the scope of the selected instruments. Although validated measures were used, these constructs were evaluated through self‐report questionnaires, which may be subject to social desirability and response biases. Given the sensitivity of mental health‐related constructs, participants may have underreported psychological symptoms or overreported resilience despite assurances of anonymity. Therefore, the findings should be interpreted as reflecting perceived mental health and coping rather than clinically diagnosed conditions. In addition, as data were collected using a single method and at a single time point, the results may be influenced by common method bias, although procedural steps such as anonymous data collection and the use of validated instruments were employed to mitigate this risk. From a data collection perspective, the use of an online survey may have introduced self‐selection bias as participation was limited to individuals with internet access and sufficient digital literacy. In addition, the absence of direct researcher supervision during data collection may have influenced response accuracy, although measures were taken to restrict access and prevent duplicate responses. Furthermore, the early versus late response bias was not formally examined, which may limit the ability to assess potential systematic differences between respondents. From an analytical perspective, the study did not account for several potential confounding variables, including prior mental health history, current use of mental health services, individual coping strategies, and recent adverse life events. These factors may have influenced the reported levels of psychological distress and resilience and should be considered when interpreting the findings. From a theoretical perspective, the study focused on a limited set of constructs (depression, anxiety, stress, and resilience), which, although central to mental health research, do not fully capture the multidimensional nature of psychological well‐being. In addition, the cross‐sectional design precludes causal inferences, meaning that the observed associations cannot be interpreted as indicating directionality or underlying mechanisms. Finally, while the study provides valuable insight into the prevalence of mental health problems among veterinarians in Türkiye, it does not address the underlying causes of these outcomes. Future research employing longitudinal designs and mixed‐methods approaches would be necessary to better understand the causal pathways and contextual factors that shape veterinarians’ mental health.

## 7. Conclusion

The present study demonstrates that mental health problems represent a significant concern among veterinarians in Türkiye, with a considerable proportion experiencing severe levels of psychological distress alongside low resilience. These findings contribute to the limited evidence base from middle‐income countries and highlight the importance of considering contextual and occupational factors in understanding mental health outcomes. Beyond documenting prevalence, the results underscore the need for multilevel interventions targeting individual, organizational, and systemic factors. Addressing mental health in the veterinary profession is critical not only to individual well‐being but also to maintaining the quality, safety, and sustainability of veterinary services.

## Funding

The open access publication fee for this article was funded by the Scientific and Technological Research Council of Türkiye (TÜBİTAK).

## Conflicts of Interest

The authors declare no conflicts of interest.

## Data Availability

The data that support the findings of this study are available from the corresponding author upon reasonable request.
